# High Plasma Homocysteine Increases Risk of Metabolic Syndrome in 6 to 8 Year Old Children in Rural Nepal

**DOI:** 10.3390/nu6041649

**Published:** 2014-04-21

**Authors:** Mohsin Yakub, Kerry J. Schulze, Subarna K. Khatry, Christine P. Stewart, Parul Christian, Keith P. West

**Affiliations:** 1 Department of International Health, Johns Hopkins Bloomberg School of Public Health, Baltimore, MD 21205, USA; E-Mails: myakub1@jhu.edu (M.Y); pchrist1@jhu.edu (P.C); kwest1@jhu.edu (K.P.W); 2 Nepal Nutrition Intervention Project-Sarlahi, Tripureswor, Kathmandu 45104, Nepal; E-Mail: skk@mos.com.np; 3 Program in International and Community Nutrition, University of California, Davis, CA 95616, USA; E-Mail: cpstewart@ucdavis.edu

**Keywords:** metabolic syndrome, homocysteine, Nepal

## Abstract

Little attention has been given to the association of plasma homocysteine (Hcy) and metabolic syndrome (MetS) in children. We have evaluated the risk of MetS with plasma Hcy in a cohort of 6 to 8 year old rural Nepalese children, born to mothers who had participated in an antenatal micronutrient supplementation trial. We assessed Hcy in plasma from a random selection of *n* = 1000 children and determined the relationship of elevated Hcy (>12.0 μmol/L) to MetS (defined as the presence of any three of the following: abdominal adiposity (waist circumference ≥ 85th percentile of the study population), high plasma glucose (≥85th percentile), high systolic or diastolic blood pressure (≥90th percentile of reference population), triglyceride ≥ 1.7 mmol/L and high density lipoprotein < 0.9 mmol/L.) and its components. There was an increased risk of low high-density lipoproteins (HDL), [odds ratios (OR) = 1.77, 95% confidence intervals (CI) = 1.08–2.88; *p* = 0.020], high blood pressure [OR = 1.60, 95% CI = 1.10–2.46; *p* = 0.015] and high body mass index (BMI) [OR = 1.98, 95% CI = 1.33–2.96; *p* = 0.001] with elevated Hcy. We observed an increased risk of MetS (OR = 1.75, 95% CI = 1.06–2.90; *p* = 0.029) with elevated Hcy in age and gender-adjusted logistic regression models. High plasma Hcy is associated with increased risk of MetS and may have implications for chronic disease later in life.

## 1. Introduction

Metabolic syndrome (MetS) is a complex disorder comprising abdominal adiposity, high-blood pressure (BP), plasma glucose (PG), dyslipidemia [high-plasma triglycerides (TG) and/or low concentrations of high-density lipoproteins (HDL)]. Insulin resistance and cardiovascular disease (CVD) have gained attention as major manifestations of the syndrome. MetS has been considered an illness of adulthood; however an increase in the prevalence of insulin resistance and MetS has been reported among children recently [1,2]. According to a systematic review by Friend *et al*., the median prevalence of MetS was 3.3% and 22% among Far East (India, South Korea and China) non-obese and obese children, respectively [3]. Given global trends toward increased adiposity, obesity and diabetes, deaths due to outcomes related to MetS, such as coronary heart disease (CHD) and type-2 diabetes, are expected to rise across the age spectrum [4].

In low income societies, the incidence in MetS among children has also been associated with a pattern of intrauterine conditions leading to low birth weight (<2500 g, LBW), rapid postnatal weight gain and less frequent breastfeeding during early life [5,6]. Given a high prevalence of LBW, rural Asian populations experiencing the nutrition transition to diets higher in fat and processed foods [7] may, therefore, be at particular risk of developing MetS. In the terai of southern Nepal, longitudinal studies have shown the incidence of LBW to be 43% [8] and the prevalence of MetS to be 11.7% at 6–8 years of age [9], with a lower risk among children whose mothers were provided antenatal folic acid supplements [9]. The research described here examines homocysteine (Hcy) as a risk factor for the MetS that was observed in this rural setting.

Homocysteine, an intermediary product of methionine metabolism, is elevated in folate and vitamin B12 deficiencies [10]. Hcy has also been accepted widely as an independent risk factor for CVD such as CHD and stroke [11,12]. Moreover, Hcy has been shown to be a thrombogenic and atherogenic substrate that potentiates atherosclerotic phenomena that may lead to adverse cardiometabolic events [13].

Despite its link with CVD, whether Hcy is more broadly associated with MetS is not well established. It is known that atherosclerosis begins in childhood and CVD can occur if children and adolescents have earlier exposure to risk factors such as elevated Hcy and components of MetS. However, to the best of our knowledge, no study has yet been done to assess the relationship of Hcy to MetS among young children living in rural South Asia. Detection of risk factors for MetS early in life in South Asian populations may suggest means to attenuate the progression of MetS and possible development of type-2 diabetes and CVD later in life [14,15].

Our hypothesis in the present study was that MetS and its components such as excess adiposity, hypertension, dyslipidemia and high glucose are associated with increased plasma Hcy concentrations in children. We addressed this question by determining the prevalence of hyperhomocysteinemia and evaluating its associations with aspects of MetS in a cohort of 6–8 year old children in rural Nepal.

## 2. Experimental Section

Children born to mothers who participated in a 5-arm trial of antenatal (to 3 months postpartum) micronutrient supplementation (1999–2001) in the rural District of Sarlahi, Nepal [8], were revisited and consented in 2006–2008 at 6–8 years of age as part of a large cohort follow-up study [9]. Procedures and primary outcomes of the original trial [8,16] and follow-up assessment [9] have been previously reported. Briefly, from 2006 to 2008, 3524 of 4130 children who were born to mothers enrolled in the micronutrient supplementation trial [8,16] were revisited in their homes to reassess vital status, height (measured by stadiometer; Harpenden, Croswell, UK), weight (measured by electronic scale; Model 881, Seca, Cambridge, MD, USA) and BP, and a 10 mL venous blood sample was collected in a heparinized tube in 3305 children, two-thirds of whom reported being fasted. For BP, the mean of last three values out of four measurements collected at one minute intervals using an automated oscillometric device (BpTRU™ BPM-300 Medical Devices Ltd., Coquitlam, BC, Canada) was taken. Standard test kits (DCA 2000 analyzer MN, Bayer HealthCare LLC, Elkhart, IN, USA) were used to estimate glycosylated hemoglobin (HbA_1c_) from whole blood. Plasma was separated in a field laboratory and total cholesterol, HDL-cholesterol, TG and glucose concentrations were measured using a Cholestech LDX analyzer (Cholestech Corp., Haywood, CA, USA). Low density lipoprotein (LDL) cholesterol was estimated using Friedewald’s formula [17]. Frozen plasma was shipped to Johns Hopkins University Bloomberg School of Public Health in liquid nitrogen for further analysis, including plasma insulin, in fasted children only, by ultrasensitive sandwich immunoassay (ALPCO Diagnostic, Salem, NH, USA). Data from these laboratory analyses have been reported previously [9].

Based on criteria of having multiple aliquots of plasma and complete data collected from the initial trial and follow up activity, 2130 children were identified as eligible for further biomarker analysis, forming the sampling frame for this analysis. This group of children was similar to the 1394 children without sufficient plasma aliquots or complete data on a range of personal and household characteristics [9]. From the 2130 children we randomly selected 1000 children (511 boys and 489 girls), balanced across maternal supplementation groups (*n* = 200 children per group evenly distributed over the duration of the field activity), for a subsequent study of micronutrient status. Hcy was measured in this subset of samples by chemiluminescent immunoassay (Immulite 1000, Siemens Diagnostics, Los Angeles, CA, USA), along with other measures of inflammation and micronutrient status [18]. Out of the 1000 participating children, 30.8% children had cholesterol, 7.2% had HDL, 6.0% had TG, and 3.2% had glucose concentrations that were below the detectable limits for the Cholestech assay. For all those who had values below the detectable limits we used the minimum detectable value of that biomarker as an estimate of biomarker concentration, allowing us to retain complete data in our analyses, but thereby overestimating the actual concentrations of those analytes for those children. LDL was not calculated when other lipid data were out of range. Also, 324 children had not adhered to fasting instructions, in whom insulin was not measured and therefore HOMA not determined. Data for lipids and glucose were analyzed from both fasted and non-fasted participants and included in the final analysis as we have reported previously [9].

Ethical clearance of the study was obtained from Institutional Review Boards at the Johns Hopkins Bloomberg School of Public Health (protocol H.22.06.05.26.A2, 20 September 2006) and the Ethics Review Committee at Institute of Medicine at Katmandu, Nepal. The study was conducted according to the principles of the Declaration of Helsinki.

### 2.1. Variable Definitions

The definition of hyperhomocysteinemia in healthy children and adolescents is not well defined in the literature. Cutoffs have varied from 8.3 to 13.75 μmol/L and differ by age and ethnicity [19,20,21,22]. In adults, hyperhomocysteinemia is commonly defined as concentrations greater than 12.0 μmol/L [23] or 15.0 μmol/L [24]. Because there is no specific cut-off for defining high plasma homocysteine level in children, population-specific 85th percentile values may guide the definition of hyperhomocysteinemia for children [22]. In this analysis the 85th percentile of Hcy was 12.6 μmol/L, close to the widely accepted cutoff for hyperhomocysteinemia in adults, and used in previously published reports [22]. Therefore, we chose a cutoff of 12.0 μmol/L to indicate hyperhomocysteinemia, despite lower cutoffs sometimes used in children, because it better reflected the risk of higher homocysteine observed in Asian populations [22] and because it is conventionally used. We have established and reported previously the cutoffs used for other components of MetS and the definition of MetS [9,25,26,27,28], which are summarized in Table 1.

**Table 1:** Variable definitions.

Variables	Definitions
Hyperhomocysteinemia	>12.0 μmol/L
Adiposity	BMI: ≥85th percentile of the entire study population (observed in *n* = 150/1000 children) Waist circumference: ≥85th percentile of the entire study population (observed in *n* = 153/1000 children)
Hypertension	Systolic blood pressure or diastolic BP ≥ 90th percentile of the U.S. reference population adjusted for age, height and sex [ 25]
Dyslipidemia	TG ≥ 1.7 mmol/L ^(^^1^^)^ HDL cholesterol < 0.9 mmol/L ^(^^2^^)^
Insulin Resistance	Homeostasis model assessment (HOMA) [to estimate insulin resistance]: Product of FPI (mU/L) and PG (mmol/L) standard factor 22.5; HOMA = (FPIxPG)/22.5 [ 28] PG: (≥85th percentile of the study population; determined in *n* = 150/1000 children)
MetS	Presence of any three of the following constituents: elevated waist circumference, high PG, high systolic or diastolic BP, high TG and low HDL [ 9]

^(1^^)^ As described by the NCEP criteria set for adults, because there is no separate recommendation for children [26]; ^(^^2^^)^ As described by the NCEP criteria for cholesterol in children and adolescents [27]; BMI, basal metabolic rate; BP, blood pressure; TG, triglyceride; HDL, high density lipoprotein; PG, plasma glucose; FPI, fasting plasma insulin; MetS, metabolic syndrome.

### 2.2. Statistical Analysis

Anthropometric measures, BP and biomarkers were examined by Hcy (≤12.0 μmol/L *vs*. >12.0 μmol/L) using independent sample t-tests. Values were expressed as mean ± SD.

The association of hyperhomocysteinemia (>12.0 μmol/L) with MetS and dichotomized individual components of MetS was expressed as odds ratios (OR) determined by separate logistic regressions for each outcome variable, with adjustment for age and gender. We also estimated OR for the combined risk of having multiple MetS components (such as low HDL and high BMI combined) against hyperhomocysteinemia through multiple logistic regression. Since we had previously shown an effect of maternal antenatal micronutrient supplementation, particularly with folic acid, on MetS in these children [9], we also initially adjusted our regression models for maternal intervention groups and birth weight, but this adjustment is not reported as these variables were not statistically significant. Likewise we examined statistical models adjusted for various aspects of socioeconomic status (SES), including ownership of televisions, radios, bicycles, livestock and use of electricity, as well as for seasonal effects. However, adjustment for these variables is not reported as their influence was not statistically significant. Finally, we explored models adjusted for fasting status and observed no effect on the OR; thus, that adjustment was not included in our results. Risks were expressed as OR and associated 95% confidence intervals (CI). All statistical analyses were done with IBM SPSS^®^ (Statistical Package for Social Sciences, IBM Corp., Armonk, NY, USA) software version 21 for Windows^®^.

## 3. Results

The prevalence of underweight, stunting and low BMI (below-2 *Z*-score) [29] in these children was 48.2%, 42.0% and 16.1% respectively. The prevalence of hyperhomocysteinemia was 18.4%. Among the 1000 children, 827 (83%) had low HDL cholesterol (<0.9 mmol/L), 108 (11%) had high triglyceride (≥1.7 mmol/L), 190 (19%) had high blood pressure (≥90th percentile of reference), 153 (15%) had high waist circumference (≥85th percentile). A total of 86 (8.6%) children met the criteria for MetS.

Table 2 indicates that children with elevated Hcy (>12.0 μmol/L) had higher BMI (*p* = 0.002), waist circumference (*p* = 0.043), systolic (*p* = 0.071) and diastolic (*p* = 0.052) blood pressure, and triglycerides (*p* = 0.016) and total lipid concentrations (*p* = 0.048). HDL concentrations were lower in subjects with higher vs. lower Hcy levels (*p* = 0.031). In logistic regression analyses adjusted for age and sex (Table 3), an elevated Hcy level was associated with an increased risk of low HDL cholesterol [OR = 1.77 (95% CI = 1.08–2.88); *p* = 0.022], high BP [OR = 1.65 (95% CI = 1.10–2.46); *p* = 0.015] and MetS [OR = 1.75 (95% CI = 1.06–2.90); *p* = 0.029]. No association was observed between elevated Hcy and high TG, waist circumference or PG.

We detected an increased risk of high BMI (≥85th percentile) [OR = 1.98 (95% CI = 1.33–2.96); *p* = 0.001] with hyperhomocysteinemia. Moreover, we observed that the relative odds of having combined low HDL cholesterol and high BP was 1.76 (95% CI = 1.14–2.70; *p* = 0.010). Similarly the odds ratio for combined low HDL cholesterol and high BMI was 2.31 (95% CI = 1.51–3.53; *p* < 0.001). We didn’t see additional risk for combined low HDL, high BP and high BMI with hyperhomocysteinemia (*p* = 0.351) (Table 3).

**Table 2:** Anthropometric measures, blood pressure, and plasma biochemical biomarkers of study children by plasma homocysteine levels ^1^.

Variables	Total *n* = 1000	Homocysteine ≤ 12.0 μmol/L *n* = 813	Homocysteine > 12.0 μmol/L *n* = 184	*p*-Value ^2^
Age	7.48 ± 0.65	7.47 ± 0.44	7.52 ± 0.40	0.24
Hcy	9.40 ± 3.50	8.06 ± 1.99	15.02 ± 2.97	<0.001
BMI ^3^	14.02 ± 1.04	13.97 ± 1.03	14.24 ± 1.05	0.002
Waist Circumference ^3^ (cm)	51.40 ± 3.06	51.31 ± 3.13	51.81 ± 2.72	0.043
Systolic BP ^3^ (mmHg)	95.2 ± 8.3	95.0 ± 8.1	96.2 ± 9.0	0.071
Diastolic BP ^3^ (mmHg)	63.8 ± 8.5	63.6 ± 8.1	64.9 ± 9.8	0.052
Total Cholesterol (mmol/L)	3.01 ± 0.48	3.01 ± 0.49	3.0 ± 0.45	0.760
TG (mmol/L)	2.55 ± 1.09	2.51 ± 1.06	2.73 ± 1.20	0.016
Total lipids (mmol/L)	5.56 ± 1.26	5.53 ± 1.22	5.73 ± 1.40	0.048
LDL (mmol/L)	1.92 ± 0.43	1.91 ± 0.45	1.92 ± 0.36	0.940
HDL (mmol/L)	0.71 ± 0.22	0.72 ± 0.23	0.68 ± 0.20	0.031
PG (mmol/L)	3.99 ± 1.06	4.00 ± 1.11	3.97 ± 0.77	0.783
FPI (pmol/L)	22.56 ± 23.76	22.62 ± 23.58	22.20 ± 25.02	0.847
HbA_1c_ (%)	5.11 ± 0.27	5.12 ± 0.28	5.09 ± 0.25	0.167

^1^ Values are means±SD; ^2^*p* compares mean values in Homocysteine (≤12.0 μmol/L) *vs*. Homocysteine (>12.0 μmol/L) groups using independent sample *t*-test; ^3^ Data were missing for BMI (*n* = 1), waist circumference (*n* = 3), systolic blood pressure (*n* = 8), diastolic blood pressure (*n* = 8), LDL (*n* = 367) and FPI (*n* = 324); BMI, body mass index; TG, triglyceride; LDL, low density lipoprotein; HDL, high density lipoprotein; PG, plasma glucose; FPI, fasting plasma insulin; HbA_1c_, glycosylate hemoglobin.

**Table 3:** Risk of MetS and its components in 6–8 years old children related to hyperhomocysteinemia (*n* = 1000) ^1^.

Outcome	Homocysteine > 12.0 μmol/L	*p*-Value
	OR (95% CI)	
MetS ^2^	1.75 (1.06–2.90)	0.029
Low HDL (<0.9 mmol/L)	1.77 (1.08–2.88)	0.022
High TG (≥1.7 mmol/L)	1.31 (0.80–2.13)	0.276
High systolic OR diastolic BP (≥90th percentile)	1.65 (1.10–2.46)	0.015
High waist circumference (≥85th percentile)	0.98 (0.63–1.53)	0.982
High PG (≥85th percentile)	1.26 (0.83–1.94)	0.275
High BMI (≥85th percentile)	1.98 (1.33–2.96)	0.001
High HOMA (≥85th percentile)	1.26 (0.73–2.16)	0.401
High BP + High BMI	1.36 (0.54–0.346)	0.512
Low HDL + High BP + High BMI	1.63 (0.58–4.60)	0.351
Low HDL + High BP	1.76 (1.14–2.70)	0.010
Low HDL + High BMI	2.31 (1.51–3.53)	<0.001

^1^ Values are OR (95% CI) based on separate logistic regression analyses for each set of outcomes, adjusted for age and gender; ^2^ MetS defined as presence of any three of the following constituents: elevated waist circumference, high PG, high systolic or diastolic BP, high TG and low HDL.

## 4. Discussion

We observed an increased risk of MetS and its components, specifically dyslipidemia, BMI, and hypertension, with elevated Hcy in young school-aged children in rural Nepal. The distributions of these components of MetS were similar to those reported previously for the larger cohort of 3524 children from whom this sample was derived [9]. That study identified intrauterine exposure to folate as a factor that ameliorated the risk of MetS in childhood; the current study expands on those findings by identifying a more proximal potential risk factor for MetS.

Previous studies have revealed that individuals who meet the criteria of MetS are at increased risk for developing CHD and diabetes mellitus [30], both of which are increasing in South Asia. It has been reported that 20%–25% of South Asians have MetS, with the frequency expected to rise in the future [31]. Nepal is not an exception, with CHD considered the leading cause of death in 2010 followed by stroke, hypertension and diabetes mellitus among other major causes of death [32]. However, data for these reports have been generated from urban areas with no study, so far, carried out to establish the burden of CVD in rural Nepal. Shaik, *et al*. collected hospital admission data in one of the tertiary hospitals which served the terai of Nepal and observed that around two patients out of ten were admitted due to stroke [33]. The extent of CVD in the region surrounding Nepal is not different, as it has been reported that 32% of all deaths in rural India are due to CVD [34].

Not limited to adults, South Asian children are also reported to have increased susceptibility to MetS and CVD, as South Asian children living in the UK showed higher mean heart rate, elevated mean triglyceride and fibrinogen levels, and higher mean fasting and post-glucose load insulin concentrations compared to white children [35]. Moreover, evidence also suggests that Indian children who are born small for gestational age have higher blood pressure, cholesterol levels (total cholesterol and LDL-C), as well as increased adiposity at the age of eight years [36]. Our results are in line with the above-mentioned studies, as we also observed high mean triglycerides and low mean HDL in these children, who were nonetheless thin (prevalence of low BMI 16%), underweight and stunted (>40% prevalence for both) [18]. Our data also suggest a role for elevated homocysteine with hypertension, dyslipidemia, and adiposity.

Although Hcy concentrations have been investigated in children in Western populations [20,37], no previous study has examined this risk factor in young South Asian children. The 95th percentile of Hcy (15.9 μmol/L), mean concentration Hcy of 9.4 μmol/L and prevalence of hyperhomocysteinemia (18.4%) observed in our study area reflect elevated levels of Hcy in rural Nepal compared to Western populations [20,37]. A high burden of hyperhomocysteinemia in children could be a risk factor for CVD in later life, as elevated Hcy (95th percentile) in Dutch children was associated with a subsequent 4-fold increased risk of ischemic cerebrovascular disease [38]. High levels of Hcy in children of rural Nepal could be due to genetic polymorphisms, environmental exposures (e.g., high blood lead), physical activity patterns, and diet, particularly one that may lead to low vitamin B12 concentrations [39].

Atherosclerosis is known to begin in childhood, as autopsy reports in children and young adults with unexpected death have revealed positive associations between atherosclerotic lesions and risk factors such as LDL-C, triglycerides, systolic and diastolic blood pressure, body mass index and cigarette smoking, making it imperative to maintain healthy lipid profiles, blood pressure [40] and plasma Hcy to minimize the burden of diseases like CVD and MetS. High levels of Hcy and high cholesterol are both associated with CVD risk. However, very few studies have reported the combined effect of hyperhomocysteinemia and dyslipidemia on the risk of CVD [41]. Since we observed a high prevalence of low HDL (83%) and considerable prevalence of hyperhomocysteinemia (18.5%) in these children, a potential interaction between Hcy and HDL cholesterol seems clinically relevant. Although exact mechanisms relating Hcy and cholesterol are not known, animal studies have revealed that hypomethylation due to hyperhomocysteinemia could be accountable for lipid accumulation in tissues [42]. Moreover, Hcy could also modulate activity of some inhibiting enzymes which play a role in HDL-particle assembly [42].

The association we observed between Hcy and blood pressure also merits discussion. We observed that an increase in Hcy was associated with rise in systolic and diastolic blood pressure, a finding consistent with National Health and Nutrition Examination Survey (1994–1998) and other studies of adult populations in developed countries [43,44]. Possible mechanisms to explain a link of Hcy with increased blood pressure are Hcy-induced arteriolar constriction [45] vascular endothelial damage [46] and decreased vasodilator responsiveness [47].

To the best of our knowledge no previous study has addressed the relationship of Hcy with MetS or components of MetS in children. Those studies which have addressed the relationship of Hcy with MetS in adults present contradictory results. Relationships we observed were similar to data published by Kang *et al*. showing a positive association between Hcy and triglycerides, BMI and systolic and diastolic blood pressure and a negative association with HDL in Korean adults [48]. Likewise, Hcy was positively associated with waist circumference, BMI, blood pressure, LDL-C, and triglycerides, but inversely associated with HDL in a Chinese sample of 1680 adults [49]. On the other hand, there are published reports where authors did not find associations [50] or observed associations with few components of MetS [51]. A lack of association of hyperhomocysteinemia with insulin resistance is consistent with other reports [52,53].

This study had some limitations. Due to the cross-sectional design we cannot establish a temporal or causal relationship between Hcy and MetS. The elevated Hcy and MetS could be results of common pathways such as poor diet, environment, inadequate physical activity [54,55], and genetic polymorphisms. Since no stringent range has been set to define hyperhomocysteinemia in children, our definition of hyperhomocysteinemia may underestimate the prevalence of hyperhomocysteinemia as the cut off >12.0 μmol/L is generally used for adults. Moreover, we somewhat overestimated the actual mean of total cholesterol, HDL, TG and plasma glucose by assigning values for these analytes equivalent to the lowest detectable concentration of these analytes, although prevalence of abnormal findings would not have been affected We believe that this study provides unique information about the high prevalence of hyperhomocysteinemia and its role as a risk factor for MetS in a population of otherwise undernourished children residing in rural Nepal. Our findings reveal important issues to be considered further on the relationship of Hcy and MetS in a population which is known to have a high burden of CVD in adulthood.

## 5. Conclusions

We conclude that hyperhomocysteinemia exerts risk towards development of MetS and that high prevalence of hyperhomocysteinemia and MetS in this low income population suggest that Nepalese children are at a greater risk of developing CVD and diabetes in future. It is therefore necessary to understand the causes of hyperhomocysteinemia, such as dietary habits in this population, in order to attenuate the future development of diabetes and cardiovascular disease symptoms, both of which constitute a growing concern among populations of South Asia.
